# Cognitive Impairment Across Kidney Replacement Therapies: A Montreal Cognitive Assessment-Based Comparative Study

**DOI:** 10.7759/cureus.114512

**Published:** 2026-08-13

**Authors:** Burak Atalay, Berfu Korucu, Serpil Muge Deger, Ege Erbay, Yelda Deligoz Bildaci

**Affiliations:** 1 Internal Medicine, Dokuz Eylül University Hospital, Izmir, TUR; 2 Nephrology, Dokuz Eylül University Hospital, Izmir, TUR; 3 Nephrology, Dokuz Eylül University Faculty of Medicine, Izmir, TUR

**Keywords:** cognitive impairment, hemodialysis, kidney transplantation, montreal cognitive assessment, peritoneal dialysis

## Abstract

Objectives

Cognitive impairment is a prevalent, often underdiagnosed complication of end-stage kidney disease. This study compared cognitive function among patients receiving hemodialysis or peritoneal dialysis and kidney transplant recipients with that of healthy controls using the Montreal Cognitive Assessment, a validated screening tool for global cognitive function. The primary outcome was the total Montreal Cognitive Assessment score, with cognitive impairment defined as a score <26.

Methods

A total of 147 participants were enrolled: 32 peritoneal dialysis patients, 31 hemodialysis patients, 42 kidney transplant recipients, and 42 healthy controls. Cognitive performance was assessed using Montreal Cognitive Assessment total and domain scores. The Montreal Cognitive Assessment was administered to hemodialysis patients before their scheduled dialysis session, whereas peritoneal dialysis patients and kidney transplant recipients were assessed during routine outpatient follow-up visits. Multivariable linear and logistic regression models identified independent predictors of cognitive performance and risk factors for impairment (score <26).

Results

Total cognitive scores were significantly lower in all patient groups than in controls; the hemodialysis group exhibited the lowest performance (20.29 ± 5.49 vs. 24.52 ± 2.98, p < 0.001). The prevalence of cognitive impairment (Montreal Cognitive Assessment score <26) was 37.5% in the peritoneal dialysis group, 48.4% in the hemodialysis group, 38.1% in the kidney transplantation group, and 15.0% among healthy controls. Hemodialysis patients performed significantly worse than kidney transplant recipients in the attention domain (p = 0.015). In the fully adjusted multivariable model, age (β = -0.120, p < 0.001) and education level (p < 0.001) were the only independent predictors of cognitive scores. Although hemodialysis patients showed a trend toward a higher prevalence of impairment, treatment modality was not an independent risk factor after adjustment for demographic factors (p = 0.123). Higher education was strongly protective, reducing the risk of impairment by 93.9% (adjusted odds ratio: 0.061, p < 0.001).

Conclusion

Cognitive impairment is common among patients with end-stage kidney disease and is likely multifactorial, with contributing factors including vascular disease, chronic inflammation, anemia, and the accumulation of uremic toxins. Although hemodialysis patients demonstrated lower cognitive performance and a higher prevalence of impairment, age and education, rather than treatment modality, were the principal independent determinants of cognitive outcomes.

## Introduction

Chronic kidney disease (CKD) is a systemic condition that leads to cardiovascular and metabolic complications while exerting detrimental effects on cognitive functions [1,2]. Compared to the general population, individuals with CKD exhibit a significantly higher prevalence of cognitive impairment [3,4]. Although the exact pathophysiology of cognitive dysfunction in CKD remains poorly understood, contributing factors include elevated uremic toxins, hypertension, anemia, and malnutrition [5]. Consequently, cognitive impairment is recognized as a major cause of morbidity and mortality in this population [6,7], suggesting that early identification and intervention may help mitigate the severity of neurological complications in advanced stages.

Currently, a consensus on the most appropriate tools for assessing cognitive functions in CKD is lacking. The literature indicates that executive functions, attention, processing speed, and memory are the most frequently affected domains [8,9]. The Montreal Cognitive Assessment (MoCA) specifically targets executive functions, which are often impaired in kidney disease [10,11]. Notably, Tiffin-Richards et al. (2014) demonstrated that MoCA is a more sensitive and specific screening tool than the Mini-Mental State Examination (MMSE) for detecting cognitive impairment in patients receiving hemodialysis [12].

However, few studies have comprehensively evaluated the cognitive impact of different kidney replacement therapy (KRT) modalities. While Shea et al. (2019) reported a 28.9% prevalence of cognitive impairment in peritoneal dialysis patients [13], Gupta et al. (2017) found cognitive deficits in 58% of kidney transplant recipients, noting that age, gender, and education level were significantly associated with performance [14].

To date, there is a lack of comprehensive research directly comparing all KRT modalities with those of healthy individuals. This study aimed to determine the prevalence of cognitive impairment among patients receiving hemodialysis, peritoneal dialysis, or kidney transplantation using the MoCA test, and to compare these findings with those in healthy individuals with normal glomerular filtration rates.

Different kidney replacement therapy modalities may influence cognitive function through distinct physiological mechanisms. Hemodialysis has been associated with recurrent intradialytic hemodynamic fluctuations and cerebral hypoperfusion, which may contribute to cognitive decline. In contrast, peritoneal dialysis provides greater hemodynamic stability and more continuous solute clearance, whereas kidney transplantation may improve cognition by restoring kidney function and reducing the burden of uremic toxins, although residual vascular and metabolic risk factors may persist. These differences provide a biological rationale for comparing cognitive outcomes across treatment modalities.

## Materials and methods

Study design

This cross-sectional study was conducted to evaluate and compare cognitive performance across different stages and modalities of chronic kidney disease (CKD) alongside healthy controls. The study was carried out at Dokuz Eylül University Hospital between March 1, 2024, and July 1, 2024.

Study participants

The study population consisted of 147 participants divided into four distinct groups: the peritoneal dialysis (PD) group (n = 32), comprising patients on active PD; the hemodialysis (HD) group (n = 31), consisting of patients undergoing active HD; the kidney transplantation (KTx) group (n = 42), including individuals with a successful history of kidney transplantation; and the control group (n = 42), composed of individuals with normal kidney function who were recruited from the internal medicine outpatient clinic during routine health evaluations. Participants with a history of dementia, prior stroke, severe psychiatric disease, active delirium, or significant visual or hearing impairment that could interfere with cognitive assessment were excluded from all groups. For all participants, sociodemographic characteristics, clinical comorbidities, and key laboratory parameters, specifically hemoglobin, estimated glomerular filtration rate (eGFR), serum electrolytes, parathyroid hormone (PTH) levels, and dialysis adequacy (Kt/V) metrics, were comprehensively documented.

Cognitive function was assessed using the validated Turkish version of the MoCA. Official institutional permission for the academic use and publication of the MoCA instrument was obtained from the copyright holders prior to the study. Cognitive impairment was defined as a MoCA score <26, according to Nasreddine et al. [15]. No education-based score correction (+1 point for participants with ≤12 years of education) was applied.

The paper-based Turkish version of the MoCA was administered face-to-face in a quiet clinical setting by the study investigators. For patients undergoing hemodialysis, cognitive assessment was performed before the dialysis session. For patients receiving peritoneal dialysis and kidney transplant recipients, the assessment was conducted during routine outpatient follow-up visits. Healthy controls completed the assessment during their routine outpatient evaluation.

Cognitive assessment and ethical considerations

The study protocol was approved by the Institutional Review Board/Ethics Committee of Dokuz Eylul University (approval date: March 27, 2024; decision no.: 2024/12-12). All participants provided written informed consent prior to enrollment, and the study was conducted in accordance with the principles of the Declaration of Helsinki.

Statistical analysis

Continuous variables were compared using Kruskal-Wallis tests with post hoc Bonferroni-corrected Mann-Whitney U tests, while categorical data were analyzed using the chi-square test. Spearman’s rank correlation (ρ) was used to identify associations between MoCA scores and clinical variables (age, eGFR, dialysis duration, and BMI). Independent predictors of cognitive outcomes and cognitive impairment risk were identified using multivariable linear and logistic regression analyses, respectively. Model performance was assessed using AUC, the Hosmer-Lemeshow test, and VIF for multicollinearity. All analyses were two-sided (p < 0.05).

## Results

Demographic and clinical characteristics

A total of 147 participants were included: peritoneal dialysis (PD, n = 32), hemodialysis (HD, n = 31), kidney transplantation (KTx, n = 42), and a healthy control group (n = 42). Significant differences were observed in age and BMI across groups (p < 0.001 for both). Post hoc analyses revealed that the KTx and control groups were significantly younger than the dialysis patients. Notably, the PD group exhibited the highest median BMI (27.2 (25.8-29.5) kg/m²) among all groups. Hypertension prevalence was significantly higher in the PD, HD, and KTx groups compared to the controls (p < 0.001). No significant differences were found regarding gender, education, smoking status, or the prevalence of diabetes and coronary artery disease (p > 0.05) (Table 1).

**Table 1:** Demographic comparisons across groups BMI: body mass index; CAD: coronary artery disease; DM: diabetes mellitus; HD: hemodialysis; HT: hypertension; IQR: interquartile range; KTx: kidney transplantation; PD: peritoneal dialysis.

Variable	(n = 32)	HD (n = 31)	KTx (n = 42)	Control (n = 42)	p-value*
Age (years), median (IQR)	61.0 (46.0-66.2)	62.0 (51.5-76.5)	49.0 (42.2-57.8)	49.0 (40.2-55.8)	< 0.001
BMI (kg/m²), median (IQR)	27.2 (25.8-29.5)	24.7 (22.8-26.7)	25.2 (23.8-27.0)	25.4 (24.3-27.0)	< 0.001
Female sex, n (%)	13 (40.6)	18 (58.1)	18 (42.9)	20 (47.6)	0.505
Education (primary/high school/university), n	13/10/9	18/9/4	14/17/11	11/18/13	0.173
Current smoker, n (%)	12 (37.5)	6 (19.4)	12 (28.6)	13 (31.0)	0.055
Diabetes mellitus	8/32 (25.0%)	11/30 (36.7%)	8/42 (19.0%)	8/42 (19.0%)	0.285
Hypertension	24/32 (75.0%)	21/31 (67.7%)	32/42 (76.2%)	12/42 (28.6%)	< 0.001
Coronary artery disease	4/32 (12.5%)	5/31 (16.1%)	8/42 (19.0%)	3/42 (7.1%)	0.431
Hyperlipidemia	14/32 (43.8%)	11/31 (35.5%)	16/42 (38.1%)	10/42 (23.8%)	0.311

Laboratory parameters revealed that phosphorus and parathyroid hormone (PTH) levels were significantly lower in the KTx group compared to both the PD and HD groups (p < 0.001). Additionally, the Kt/V ratio was significantly higher in the PD group than in the HD group (2.04 (2.00-2.30) vs. 1.70 (1.50-2.00), p < 0.001). No significant difference was observed in dialysis duration between the two dialysis modalities (36.0 (12.0-48.0) vs. 17.0 (8.0-45.0) months, p = 0.316). Detailed laboratory findings are presented in Table 2. 

**Table 2:** Detailed laboratory findings HD: hemodialysis; IQR: interquartile range; Kt/V: dialysis adequacy ratio (K: dialyzer clearance of urea; t: dialysis time; V: volume of distribution of urea); PD: peritoneal dialysis; PTH: parathyroid hormone; KTx: kidney transplantation.

Parameter	PD Median (IQR)	HD Median (IQR)	KTx Median (IQR)	P
Hemoglobin (g/dL)	11.7 (10.7-12.5)	11.2 (10.0-12.3)	11.9 (11.0-12.7)	0.133
Sodium (mmol/L)	139.0 (137.0-140.0)	138.0 (137.0-139.5)	138.0 (136.0-140.8)	0.737
Potassium (mmol/L)	4.0 (3.8-4.3)	4.6 (3.9-4.8)	4.0 (3.7-4.7)	0.096
Calcium (mg/dL)	8.6 (8.4-8.9)	8.5 (8.3-8.7)	8.6 (8.5-8.7)	0.264
Phosphorus (mg/dL)	5.1 (4.7-6.0)	4.8 (4.6-5.7)	4.1 (3.8-4.7)	<0.001
PTH (pg/mL)	340.5 (289.2-427.5)	350.0 (216.0-489.5)	107.5 (81.8-137.5)	<0.001
Kt/V ratio	2.04 (2.00-2.30)	1.70 (1.50-2.00)	—	< 0.001
Dialysis duration (months)	36.0 (12.0-48.0)	17.0 (8.0-45.0)	—	0.316

Comparison of MoCA total and domain scores

The MoCA total scores differed significantly across the study groups (p < 0.001). The highest median score was observed in the healthy control group (25.0 (23.0-27.0)), while the lowest was observed in the HD group (21.0 (15.0-24.5)). Median scores for the PD and KTx groups were identical (23.0 (19.0-26.0) for both) (Table 3).

**Table 3:** MoCA total score comparison across groups HD: hemodialysis; IQR: interquartile range; Min–Max: minimum–maximum values; PD: peritoneal dialysis; KTx: kidney transplantation; SD: standard deviation; MoCA: Montreal Cognitive Assessment.

Group	N	Mean±SD	Median (IQR)	Min-Max
PD	32	22.62±4.48	23.0 (19.0-26.0)	10-29
HD	31	20.29±5.49	21.0 (15.0-24.5)	11-29
KTx	42	21.95±5.01	23.0 (19.0-26.0)	10-30
Control	42	24.52±2.98	25.0 (23.0-27.0)	17-29

Detailed domain analysis using Bonferroni-corrected Mann-Whitney U tests revealed specific cognitive deficits (Table 4). Compared to the healthy controls, the HD group showed significantly lower scores in the visuospatial (p = 0.029), memory (p = 0.002), and attention (p = 0.026) domains. Furthermore, the HD group performed significantly worse in the attention domain than the KTx group (p = 0.015). Notably, all three patient groups (PD, HD, and KTx) exhibited significantly lower verbal fluency scores compared to the control group (all p < 0.001). 

**Table 4:** Significant group comparisons based on Bonferroni-corrected Mann–Whitney U tests HD: hemodialysis; PD: peritoneal dialysis; KTx: kidney transplantation; U: Mann–Whitney U statistic.

Domain	Significant comparison	U	Bonferroni p
Visuospatial	HD – Control	407.0	0.029
Memory	HD – Control	356.5	0.002
Attention	HD – KTx	391.5	0.015
	HD – Control	403.0	0.026
Verbal fluency	PD – Control	275.0	<0.001
	HD – Control	334.0	<0.001
	KTx – Control	441.0	<0.001

Correlation and regression analyses

Spearman correlation analysis demonstrated that MoCA total scores were strongly and negatively correlated with age (ρ = −0.646, p < 0.001). Weak positive correlations were identified between MoCA scores and hemoglobin levels (ρ = 0.233, p = 0.005) and dialysis duration (ρ = 0.315, p = 0.029) (Table 5). In the fully adjusted multivariable linear regression model, age (B = −0.120, 95% CI: −0.158 to −0.082, p < 0.001) and education level were identified as the strongest independent predictors of MoCA scores. Compared to primary school education, having a high school education (β = 3.389, 95% CI: 2.075-4.703, p < 0.001) or a university education (β = 5.907, 95% CI: 4.376-7.439, p < 0.001) was associated with significantly higher cognitive performance. Notably, after adjusting for age and education, dialysis modality and transplantation status did not reach statistical significance as independent predictors of cognitive function (Table 6). 

**Table 5:** Correlations between MoCA total score and continuous variables BMI: body mass index; eGFR: estimated glomerular filtration rate; PTH: parathyroid hormone; MoCA: Montreal Cognitive Assessment.

Variable	Valid n	Spearman ρ	Spearman p
Age	147	-0.646	< 0.001
Hemoglobin	147	0.233	0.005
eGFR	40	0.212	0.189
Dialysis duration (months)	48	0.315	0.029
PTH	105	-0.032	0.750
BMI	147	0.032	0.700

**Table 6:** Multivariable linear regression analysis for MoCA scores BMI: body mass index; CAD: coronary artery disease; CI: confidence interval; DM: diabetes mellitus; HD: hemodialysis; HT: hypertension; KTx: kidney transplantation; MoCA: Montreal Cognitive Assessment; PD: peritoneal dialysis; PTH: parathyroid hormone.

Variable	β	95% CI	p-value
Intercept	32.762	25.736–39.787	<0.001
Age	-0.120	-0.158-0.082	<0.001
Sex (male)	0.238	-1.063–1.539	0.719
Education (high school)	3.389	2.075–4.703	<0.001
Education (University)	5.907	4.376–7.439	<0.001
Group (PD)	-0.355	-2.251–1.540	0.710
Group (HD)	-1.770	-3.839–0.300	0.093
Group (KTx)	-1.110	-2.849–0.629	0.211
Hemoglobin	0.237	-0.179–0.653	0.261
Phosphorus	-0.214	-0.633–0.205	0.313
PTH	-0.001	-0.003–0.002	0.599
BMI	0.004	-0.158–0.166	0.960
DM	-0.723	-2.241–0.795	0.347
HT	-0.777	-2.219–0.665	0.288
CAD	-1.818	-4.046–0.410	0.109

Risk factors for cognitive impairment

To identify the independent risk factors for cognitive impairment (defined as MoCA <26), a multivariable logistic regression model was used (Table 7). Each one-year increase in age was associated with a 9.4% increase in the risk of cognitive impairment (aOR: 1.094, 95% CI: 1.062-1.128, p < 0.001).

**Table 7:** Multivariable logistic regression model for cognitive impairment (MoCA < 26) aOR: adjusted odds ratio; BMI: body mass index; CAD: coronary artery disease; CI: confidence interval; DM: diabetes mellitus; HD: hemodialysis; HT: hypertension; KTx: kidney transplantation; MoCA: Montreal Cognitive Assessment; PD: peritoneal dialysis; PTH: parathyroid hormone.

Variable	aOR	95% CI	P
Age (years)	1.094	1.062-1.128	<0.001
Sex (male)	1.186	0.520-2.705	0.686
Education (high school)	0.238	0.096-0.587	0.002
Education (University)	0.061	0.019-0.197	<0.001
Group (PD)	1.562	0.493-4.952	0.450
Group (HD)	2.437	0.789-7.523	0.123
Group (KTx)	1.744	0.594-5.119	0.311
DM	1.307	0.508-3.361	0.578
HT	1.498	0.606-3.704	0.386
CAD	2.057	0.637-6.643	0.229
Hemoglobin	0.918	0.761-1.107	0.369
BMI	0.998	0.900-1.107	0.967

Conversely, higher education levels served as strong protective factors against cognitive impairment. Compared to participants with only primary education, university graduates had a 93.9% lower risk of cognitive impairment (aOR: 0.061, 95% CI: 0.019-0.197, p < 0.001), while high school graduates had a 76.2% lower risk (aOR: 0.238, 95% CI: 0.096-0.587, p = 0.002).

Although the HD group showed a trend toward a higher risk of cognitive impairment (aOR: 2.437, 95% CI: 0.789-7.523), this association did not reach statistical significance in the fully adjusted model (p = 0.123). Similarly, comorbidities such as diabetes, hypertension, and coronary artery disease were not found to be independent predictors of cognitive impairment in this cohort (p > 0.05). 

## Discussion

This study evaluated the cognitive profiles of patients across different KRT modalities using the MoCA scale. Our findings showed that patients undergoing HD tended to exhibit lower cognitive performance, particularly in attention, memory, and visuospatial domains. Although the mean MoCA scores of KTx and PD patients were numerically higher than those of the HD group, these differences were not statistically significant. However, all patient groups demonstrated significant deficits in verbal fluency compared with healthy controls. Notably, multivariable analyses demonstrated that age and educational level were the primary independent predictors of cognitive impairment rather than the treatment modality itself.

The relatively high prevalence of cognitive impairment observed in HD patients in our cohort is consistent with the findings of Khrulev et al. (2019), who reported substantial cognitive decline in this population [16]. However, the rates observed in our study were lower than those reported by Gupta et al. (2017) [14], which may be explained by the relatively younger age of our study population. In our cohort, HD patients performed significantly worse in attention and memory domains compared with the KTx and PD groups (p < 0.05). These findings highlight how HD-related physiological factors, such as rapid fluid and electrolyte shifts, intradialytic cerebral hypoperfusion, and chronic systemic inflammation, may contribute to neurocognitive vulnerability in this population. These mechanisms likely reflect both acute intradialytic effects, such as transient reductions in cerebral perfusion during dialysis sessions, and chronic cumulative vascular and inflammatory injury. However, because of the cross-sectional design, our study cannot distinguish between these acute and chronic effects. These observations are consistent with the findings of Bugnicourt et al. (2013), who described executive and attentional dysfunction as common features among patients undergoing HD [17].

A notable finding of our study was the consistent decline in verbal fluency across all KRT modalities (PD, HD, and KTx) compared with healthy controls. Our results indicate that verbal fluency, which requires both executive functioning and linguistic processing, is particularly sensitive to the cognitive burden associated with CKD. In addition to these executive deficits, memory-related impairments were also prominent among patients receiving dialysis therapy. As discussed by Murray et al. (2006), the specific vulnerability of the hippocampus to ischemic stress and metabolic fluctuations may provide a mechanistic explanation for the memory deficits observed in this population [18].

The numerically higher cognitive scores observed in KTx and PD patients compared with HD patients may reflect both physiological and selection-related factors. In KTx recipients, improvements in uremic toxin clearance, stabilization of blood pressure, and correction of metabolic and endocrine abnormalities may contribute to better neurological outcomes. In PD patients, the continuous nature of dialysis may reduce the large metabolic fluctuations commonly observed during intermittent HD sessions. In addition, selection bias may have contributed to these findings, as PD is often offered to patients with better functional capacity and higher levels of self-management.

Although unadjusted MoCA scores differed numerically between treatment groups, the effect of treatment modality was no longer statistically significant after adjustment for age and educational level. This finding should not be interpreted as evidence that treatment modality has no influence on cognitive function. Rather, in our cohort, the potential effect of treatment modality appeared to be outweighed by demographic characteristics, particularly age and educational attainment. Furthermore, the relatively modest sample size may have limited our ability to detect subtle independent effects of treatment modality. These findings suggest that patient-related characteristics may have a greater influence on cognitive performance than the specific dialysis modality itself. The strong protective effect of education supports the concept of cognitive reserve [19], which proposes that higher educational attainment may mitigate the clinical manifestations of neurological damage. Similar observations emphasizing the importance of demographic factors have also been reported by Khrulev and Gupta [14,16]. From a clinical perspective, these findings support routine cognitive screening, particularly among older patients and those with lower educational attainment, who may benefit from earlier recognition of cognitive impairment and targeted educational or rehabilitative interventions.

Contrary to previous reports suggesting that longer dialysis duration is associated with worse cognitive outcomes [20], our study demonstrated a weak positive correlation between dialysis duration and MoCA scores. This unexpected finding should be interpreted cautiously. It may reflect survivor bias, whereby patients who remain on long-term dialysis represent a healthier subgroup with greater physiological resilience. Alternatively, greater engagement with healthcare services and adaptation to dialysis over time may contribute to better cognitive performance. Residual confounding by unmeasured factors also cannot be excluded, and therefore no causal relationship between dialysis duration and cognitive performance can be inferred from our findings.

This study has several limitations that should be acknowledged. First, its cross-sectional design precludes causal inference regarding the relationship between kidney replacement therapy modalities and cognitive outcomes. Although treatment modality was not independently associated with cognitive performance after adjustment, this finding does not exclude a potential effect of treatment modality. Rather, any true effect may have been attenuated by demographic factors or limited by the sample size. Second, the relatively small sample size within each subgroup may have limited the statistical power to detect subtle differences between treatment modalities, increasing the possibility of a type II error. Third, although we adjusted for major confounders such as age and educational level, residual confounding from unmeasured variables, including socioeconomic status, depression, medication use, and detailed vascular risk profiles, cannot be excluded. Furthermore, this was a single-center study conducted in a Turkish tertiary care hospital, which may limit the generalizability of our findings to populations with different ethnic backgrounds, healthcare systems, and educational structures.

Additionally, cognitive function was assessed using a single screening tool (MoCA). Although MoCA is a sensitive and well-validated instrument for detecting cognitive impairment, it does not provide a comprehensive neuropsychological evaluation across specific cognitive domains, such as processing speed, executive function, visuospatial abilities, and memory. Consequently, subtle domain-specific deficits may not have been fully characterized. Another limitation is the lack of longitudinal follow-up, which precludes assessment of changes in cognitive function over time and the potential dynamic effects of different treatment modalities. Finally, selection bias may have influenced the results, particularly in the peritoneal dialysis and transplant groups, where patients generally have better functional status and greater capacity for self-management.

Despite these limitations, this study has several important strengths. To our knowledge, this is one of the few studies to directly compare cognitive performance across all major kidney replacement therapy modalities, including hemodialysis, peritoneal dialysis, and kidney transplantation, alongside a healthy control group within the same study framework. The use of a standardized and clinically validated cognitive assessment tool (MoCA) enabled evaluation of both global and domain-specific cognitive performance. Furthermore, multivariable regression analyses strengthened the robustness of our findings by identifying independent predictors of cognitive outcomes while minimizing the influence of confounding variables.

Another key strength is the comprehensive assessment of clinical, laboratory, and demographic variables, enabling a multidimensional evaluation of factors associated with cognitive impairment. Importantly, our findings highlight the dominant role of patient-related factors, particularly age and educational attainment, in cognitive performance, further supporting the concept of cognitive reserve in CKD populations. Overall, our results support the implementation of routine cognitive screening in patients with CKD, especially among older individuals and those with lower educational attainment. They also suggest that decisions regarding kidney replacement therapy should continue to be guided primarily by overall clinical indications, while recognizing that optimization of cardiovascular risk factors, patient education, and cognitive support may have a greater impact on long-term cognitive health than treatment modality alone.

## Conclusions

This study evaluated the prevalence of cognitive impairment in individuals with CKD receiving different KRT modalities using the MoCA test and compared the findings with those from healthy individuals. The results indicate that cognitive impairment is common among individuals with CKD and particularly affects executive functions such as memory, attention, and trail-making. Older age and lower educational attainment were significantly associated with cognitive function in individuals with CKD, supporting the use of screening tools to facilitate early detection of cognitive deficits in clinical practice.
